# New Method for Subcutaneous Morphia

**Published:** 1874-02

**Authors:** 


*EDINBURGH MEDICAL JOURNAL—December,* 1873:

New and Easy Method for Sub-cutaneous Morphia. — Dr. John M. Crombie believes that many medical men are deterred from using sub-cutaneous morphia, because of expense of the syringe, need of frequent repair, and patients object to the pain. "His method of avoiding these misfortunes is to use small threads coated with the requisite quantity of morphia, which are inserted by means of fine needles, into a fold of skin, pinched up between the finger and thumb. Theoretically, the plan is ingenious and excellent. The practical difficulty that we would fear is, the risk of the morphia either not getting into the wound, by being rubbed off the thread or being so well incorporated with the thread as not to be dissolved out of it during the time it is retained, which is to be from three to four minutes. The suppositories, as Dr. Crombie calls the threads, vary in strength from one-sixth, one-quarter, one-half to one grain of morphia. The larger ones are not often necessary."

